# Clinical diagnostic value of long non-coding RNAs in Colorectal Cancer: A systematic review and meta-analysis

**DOI:** 10.7150/jca.46358

**Published:** 2020-07-11

**Authors:** Bi Chen, Ruo Nan Zhang, Xingxing Fan, Jue Wang, Cong Xu, Bo An, Qiao Wang, Jing Wang, Elaine Lai-Han Leung, Xinbing Sui, Qibiao Wu

**Affiliations:** 1 Faculty of Chinese Medicine, Macau University of Science and Technology, Taipa, Macau, P.R. China.; 2 State Key Laboratory of Quality Research in Chinese Medicines, (Macau University of Science and Technology), Taipa, Macau, P. R. China.; 3 Department of Medical Oncology, Holistic Integrative Oncology Institutes and Holistic Integrative Cancer Center of Traditional Chinese and Western Medicine, the Affiliated Hospital of Hangzhou Normal University, College of Medicine, Hangzhou Normal University, Hangzhou, Zhejiang, China.; 4 Department of Cancer Pharmacology, Holistic Integrative Pharmacy Institutes, College of Medicine, Hangzhou Normal University, Hangzhou, Zhejiang, China.

**Keywords:** long non-coding RNAs, colorectal cancer, diagnosis, systematic review

## Abstract

**Background:** Histopathological diagnosis remains the gold standard for the diagnosis of cancer, including colorectal cancer, but it is infeasible when tumor tissue is not available. With the recognition of long non-coding RNAs (lncRNAs), the expression of lncRNAs in serum or tissue samples has been reported as a diagnosis method for some cancers, however, the diagnostic value of lncRNAs for colorectal cancer remains unclear.

**Methods:** A systematic review and meta-analysis were conducted. Eligible studies were identified through a comprehensive literature search in PubMed, PubMed Central, Web of Science, Embase, and Cochrane Library (up to May 05, 2020) according to the selection criteria. Meta-DiSc, Review Manager and STATA were used to analyze the association between lncRNAs expression and the diagnosis of colorectal cancer.

**Results:** Fifteen studies that analyzed the expression of 15 lncRNAs in 1434 CRC patients were included. The summary area under the curve (AUC) of lncRNA for the diagnosis efficacy between patients with and without CRC was estimated to be 0.8629, corresponding to a weighted sensitivity of 0.75 (95% CI: 0.72 - 0.77), specificity of 0.80 (95%CI: 0.78 - 0.82). Subgroup analysis illustrated that the AUC of blood-based detection of lncRNA showed 0.8820, pooled DOR: 18.57, while tissue-based analysis showed 0.8203, pooled DOR: 10.47. Blood-based tests were then divided into two categories, plasma-based and serum-based lncRNA testing. Results revealed that the AUC of serum-based detection was 0.9077, pooled DOR: 26.64, and plasma-based detection was 0.5000, pooled DOR: 11.80.

**Conclusions:** This meta-analysis indicates that the aberrantly expressed lncRNAs might serve as potential diagnostic biomarkers for CRC patients and blood-based lncRNA analysis is of higher diagnostic accuracy than tissue-based testing. Moreover, serum-based lncRNA testing achieved higher diagnostic efficacy than plasma-based analysis.

## Introduction

Based on the GLOBOCAN2018 evaluation criteria for cancer morbidity and mortality by IARC, colorectal cancer (CRC) is still the third most commonly diagnosed malignancy with the second most lethal cancer rate worldwide 1. According to the National Cancer Statistics released by the National Cancer Center in 2019, the incidence of colorectal cancers (CRC) ranks fourth among malignant tumors and has become the fifth leading cause of cancer mortality in China 2. Previous studies have indicated that the colorectal cancer patients in the early stage would gain longer survival time with standard treatment 3-5. The prognosis of CRC can be improved when the patients are identified at their early stages. Therefore, we are supposed to improve CRC diagnostic strategies, which is as important as discovering new treatments.

Currently, the main biomarkers of colorectal cancer in clinical practice consist of carcinoembryonic antigen (CEA), cancer antigen 19-9 (CA19-9) and cancer antigen 242 (CA242). But the sensitivity and specificity are far from satisfying clinical needs. A previous study has demonstrated that the sensitivity of CEA was 46.59% and its specificity was 80% 6. Another study using ROC curves to comparing the specificity and sensitivity of CEA and CA19-9 in the early diagnosis of CRC, the AUC of CEA is 0.797 (P < 0.001) and the AUC of CA19-9 is 0.664 (P = 0.001) 7. There is a pressing need for more ideal biomarkers with higher sensitivity and specificity for early diagnosis of CRC.

Long non-coding RNAs (lncRNAs) are defined as capped transcripts > 200 nucleotides 8. It is reported that several lncRNAs are aberrantly expressed in tissue or serum from colorectal cancer patients. Some studies have reported that MEG3 9, lnc-ATB 10 and BLACAT1 11 were up-expressed in colorectal cancer plasma, while NKILA 7 and HOTAIRM1 12 were down-regulated in colorectal cancer tissue. A sensitivity of 37.16% and a specificity of 88.75% for CEA were significantly lower than that for exosomal CRNDE-h (*P* < 0.001) 13. NKILA exhibited relatively higher sensitivity and specificity compared with CEA and CA19-9 in the early diagnosis of CRC 7. And LINC02418 was also more sensitive than those existing detection methods 14.

Studies based on LncRNAs detection are constantly springing up; however, the diagnostic value of lncRNAs for colorectal cancer remains unclear. So we performed a meta-analysis to evaluate the relationship between clinical outcomes (clinicopathological parameters, diagnosis) and the expression of lncRNAs in patients with colorectal cancer, hoping to provide a theoretical basis for clinical application.

## Materials and Methods

### Literature Search

Two of the authors (Bi Chen and Ruonan Zhang) each searched several databases, including PubMed, PubMed Central (PMC), Web of Science, Embase, and Cochrane Library. The publication data used in the literature search was from database inception to May 05, 2020. The search strategies were based on combinations of the following keywords in titles or abstracts: (lncRNA OR long ncRNA OR lincRNA OR long non-coding RNA OR long non-translated RNA OR long untranslated RNA OR long non-protein-coding RNA OR long intergenic non-protein coding RNA) and (colorectal cancer OR colorectal neoplasm OR colorectal tumor OR colorectal carcinoma) and (“diagnose” or “diagnosis”).

### Selection Criteria

All included studies met the following inclusion criteria: 1) patients were diagnosed with CRC by histopathology and without other tumors; 2) volunteers were defined as no tumor and other diseases; 3) the expression level of lncRNAs was identified and analyzed for the diagnosis of CRC, and; 4) studies contained sufficient data, including sensitivity (SEN), specificity (SPE), area under the curve (AUC) or receiver operating characteristic (ROC). If unavailable, related data obtained by contacting the corresponding authors.

Exclusion criteria were: 1) duplicated study; 2) conference abstract, review, meta-analysis, animal or cellular study; 3) unrelated to CRC or lncRNA; 4) incomplete or uncorrected study.

### Data Extraction

Two independent authors (Bi Chen and Ruonan Zhang) collected the following information from the literature for each publication: (1) basic information: first author, publication year, type of lncRNA, expression, detection sample, test method, cut-off value, number of patients; (2) clinicopathological information: P values of age, gender, tumor location, tumor size, differentiation, lymphatic metastasis, distal metastasis, TNM stage, CEA level, CA 19-9 level and depth of invasion; (3) diagnosis information: SEN, SPE, AUC, ROC; (4) Engauge Digitizer software was utilized to obtain the SEN and SPE from ROC when data was not reported directly.

### Quality Assessment

Study quality was assessed with the Quality Assessment of Diagnostic Accuracy Studies II (QUADAS-2) checklist, which is recommended for use in systematic reviews and applicability of primary diagnostic accuracy studies, consisting of four key domains covering patient selection, index test, reference standard, flow and timing. Each is assessed in terms of risk of bias and the first three in terms of concerns regarding applicability 15.

### Statistical Analysis

The Meta-DiSc software (Version 1.4) was used to calculate the combined sensitivity, combined specificity, positive likelihood ratio (PLR), negative likelihood ratio (NLR), overall diagnostic odds ratio (DOR), diagnostic advantage and 95% confidence interval for each study 16. Review Manager (Version 5.3. Copenhagen: The Nordic Cochrane Centre, The Cochrane Collaboration, 2014) and STATA 15.0 software (StataCorp, College Station, TX 77845, USA) were used to analyze study data and construct the forest plot. Heterogeneity among studies was assessed by the Cochran's Q and the *I^2^* statistic. The fixed-effects model was used if there was no substantial heterogeneity (*P* > 0.10 or *I^2^* < 50%). Inversely, the random-effects model was chosen when significant heterogeneity was observed 17-19. Associations between lncRNA expression and clinicopathologic parameters were determined using the *P* values combined with Fisher's test. Publication bias was quantitatively judged by Deeks' funnel plot asymmetry test 20. *P* < 0.05 was considered to be statistically significant.

## Results

### Study Selection

**Figure 1:**
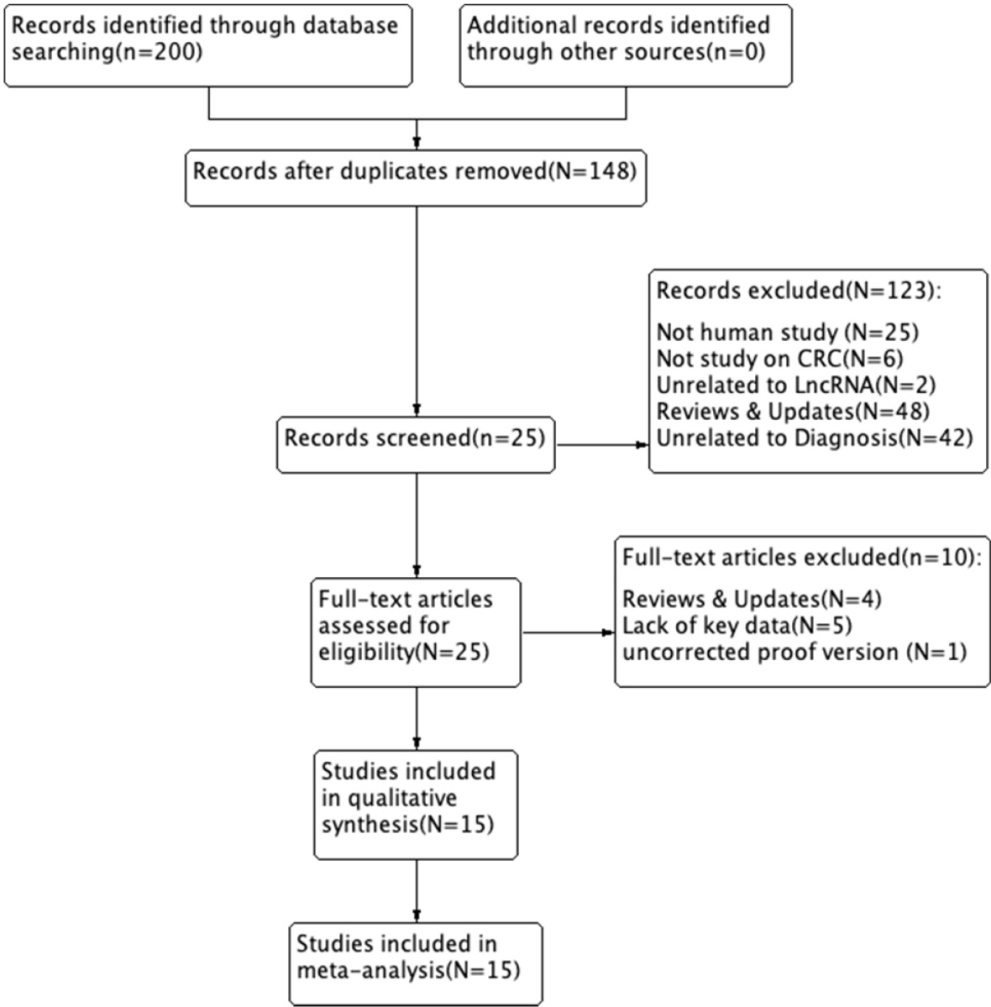
Flow diagram of the study selection process.

We searched and captured 200 records in PubMed, PMC, EMBASE, Web of Science, and the Cochrane Library. Of these, 52 duplicate studies were excluded. We excluded 123 records after reading the titles and abstracts. Subsequently, we assessed the remaining 25 full-text articles and excluded 10 studies based on the exclusion criteria, including 4 reviews & updates, 5 lacking key data, and 1 uncorrected proof version. A total of 15 studies were ultimately included in this study. A flow diagram of the selection process for this study is presented in *Figure 1*.

### Study parameters and study quality

Eighty-five percent of the selected studies were from China, with 11/15 (73.3%) being published between 2018 and 2020. A total of 1434 CRC patients were included and the number of patients ranged from 34 to 174. All patients were diagnosed based on the histopathological diagnostic criteria. The tissue samples and blood samples (serum and plasma samples) were obtained prior to clinical treatment. The expression levels of LncRNAs were determined using quantitative real-time polymerase chain reaction (qRT-PCR). Seven types of lncRNAs were recognized as tumor promoters 10, 11, 13, 14, 21-25 and six were tumor suppressors 7, 9, 26-29 in CRC patients. The associations between lncRNAs and clinicopathologic parameters in CRC patients are shown in *Table 1*. Diagnostic accuracy differed greatly between different lncRNAs. We found that ENST00000455974 detection had the highest sensitivity (95.6%) with a specificity of 81.2% 23 and CRNDE-h detection had the highest specificity (91.5%) with a sensitivity of 70.3% 13.

**Figure 2:**
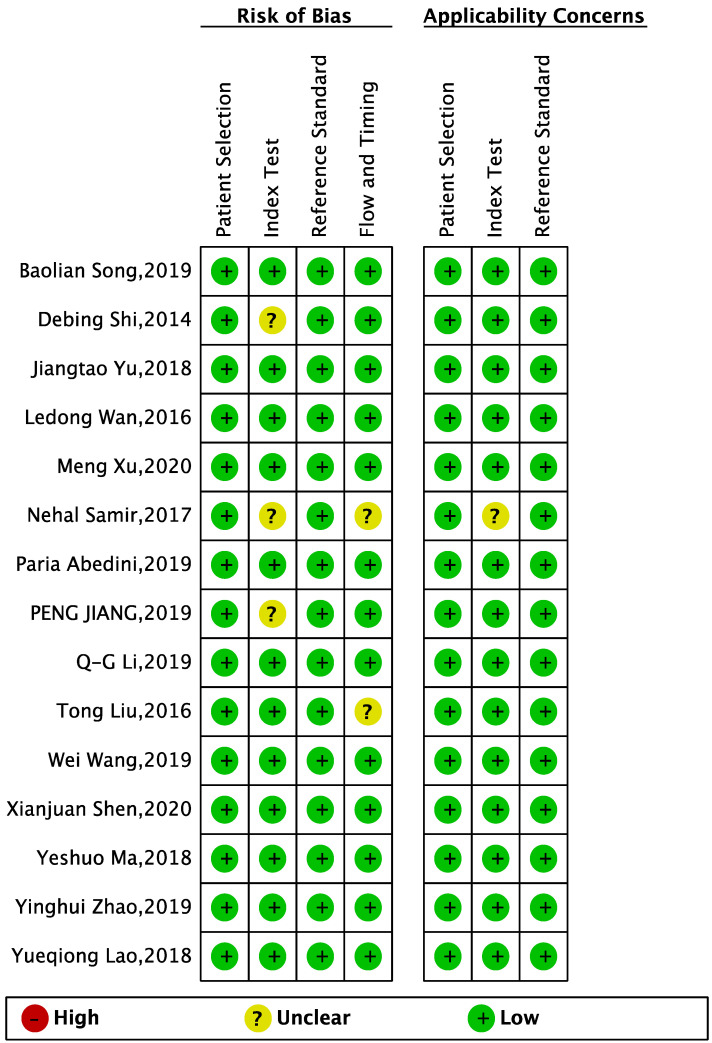
Methodological quality graph.

**Figure 3:**
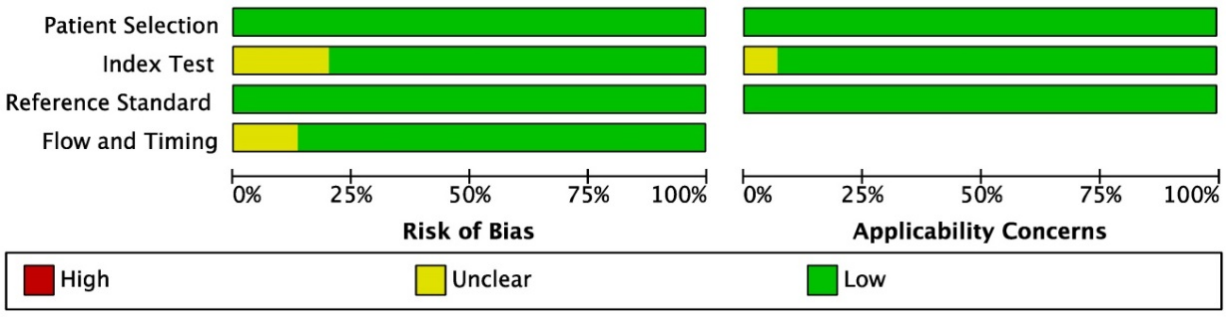
Methodological quality summary.

QUADAS-2 checklist was used to systematically assess the quality of all the included studies. The results indicated that all included studies were of high methodological quality. The results are shown in *Figures 2-3*.

### Meta-analysis of clinicopathological parameters

The *P* values between different lncRNAs and clinicopathological parameters in CRC patients of all included studies are summarized in *Table 2*. Some clinicopathological parameters were not observed or reported in some studies, so the relevant data were not available. Altered expression of lncRNAs were significantly associated with some clinicopathological parameters we collected (tumor size: pooled *P* < 0.0001; lymphatic metastasis: pooled *P* < 0.0001; TNM stage: pooled *P* < 0.0001; levels of CEA: pooled *P* = 0.0007). But, it was not correlated with age (pooled *P* = 0.5507), gender (pooled *P* = 0.2558), or tumor location (pooled *P* = 0.7519).

### Diagnostic performance

**Figure 4:**
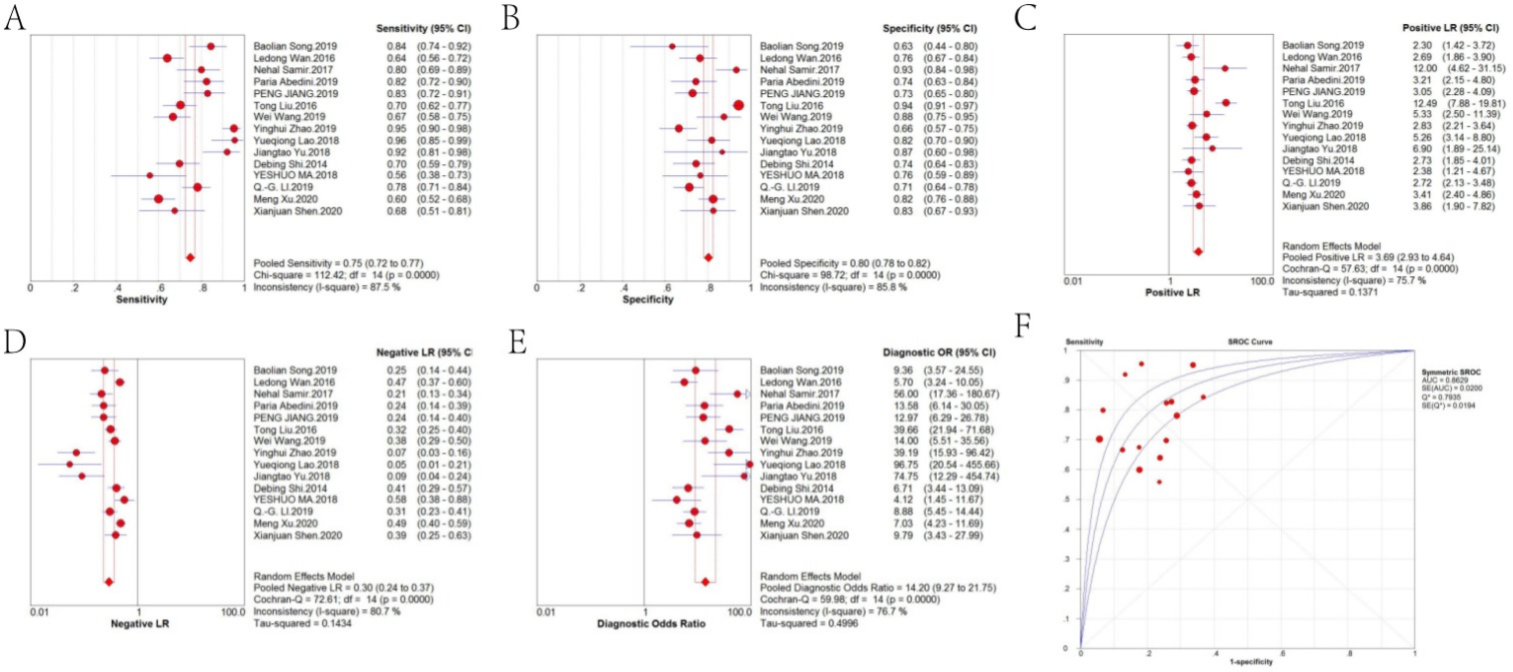
Forest plot of the (A) pooled sensitivity, (B) pooled specificity, (C) pooled PLR, (D) pooled NLR, (E) pooled DOR and (F) sROC curve of lncRNAs for the diagnosis of CRC.

Forest plots of the pooled sensitivity, pooled specificity, pooled DOR and sROC curve of lncRNAs in diagnosing CRC are shown in *Figure 4*. The weighted diagnostic parameters of lncRNAs in distinguishing CRC from non-tumor controls are as follows: pooled sensitivity of 0.75 (95%CI: 0.72 - 0.77), pooled specificity of 0.80 (95%CI: 0.78 - 0.82), pooled PLR of 3.69 (95%CI: 2.93 - 4.64), pooled NLR of 0.30 (95%CI: 0.24 - 0.37), pooled DOR of 14.20 (95%CI: 9.27 - 21.75). The AUC of the sROC curve based on summary sensitivity and specificity was 0.86.

### Meta-regression analysis

Due to the significant heterogeneity between these studies observed in sensitivity and specificity data (*I*^2^ = 87.50% and *I*^2^ = 85.80%, respectively), we firstly performed an analysis of diagnostic threshold showed in *Table* 3 to reveal no threshold effect in the studies (Spearman correlation coefficient: 0.314, *P* = 0.254). Then we constructed a meta-regression analysis in terms of the specified covariates including sample size, sample types and sample expression of lncRNAs. According to the *P* value from large to small, "size" and "expression" were eliminated one by one, and meta-regression analysis was performed. The results in *Tables 4-6* showed that the heterogeneity might be associated with the sample type (RDOR = 1.69, 95%CI: 1.05 - 2.72, *P* = 0.0339). The detection accuracy of blood samples was 1.18 times higher than that of non-blood samples, as shown in *Table 6*.

**Figure 5:**
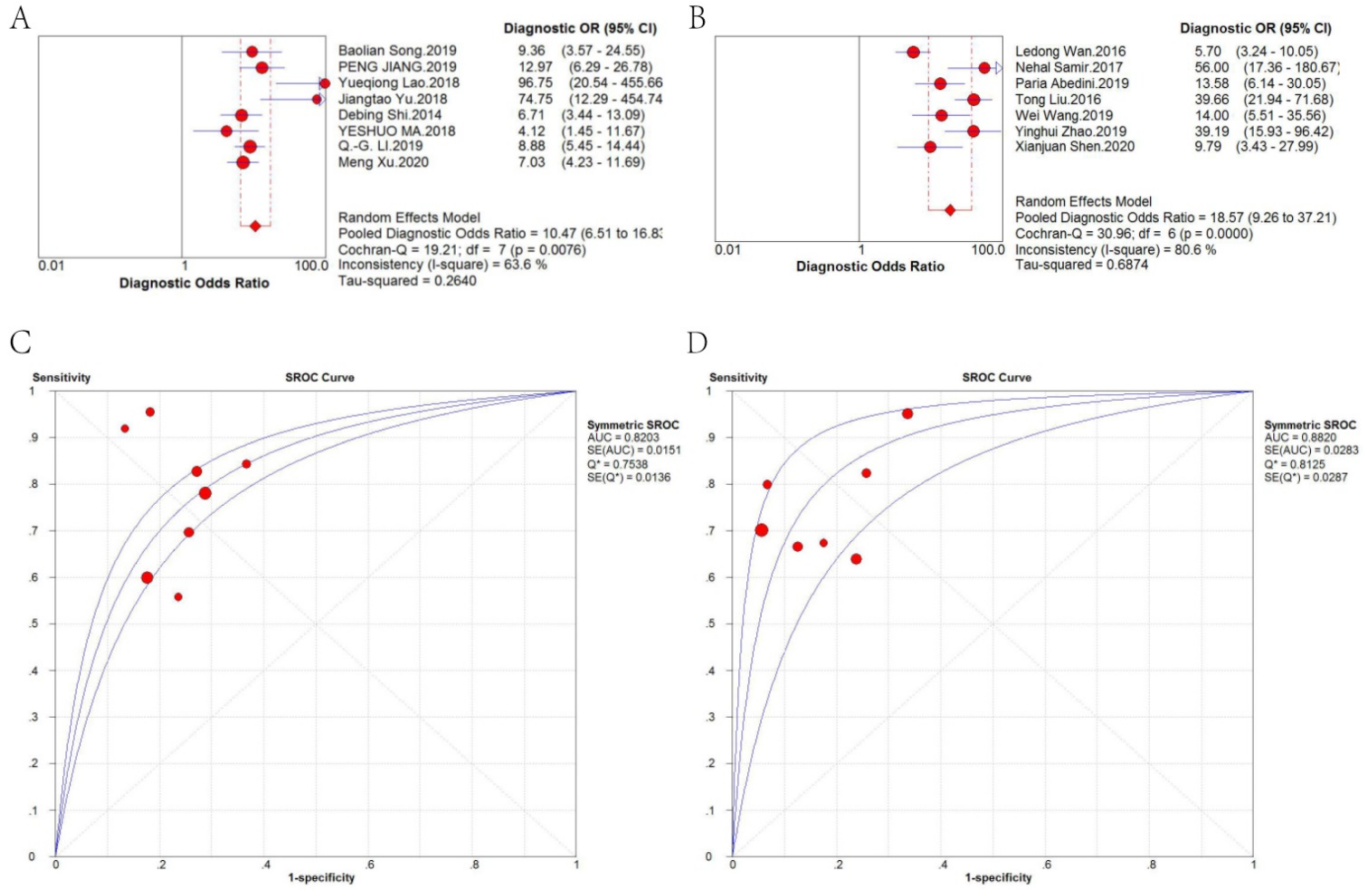
DOR and sROC of (A) tissue-based and (B) blood-based detection of lncRNAs for the diagnosis of CRC.

**Figure 6:**
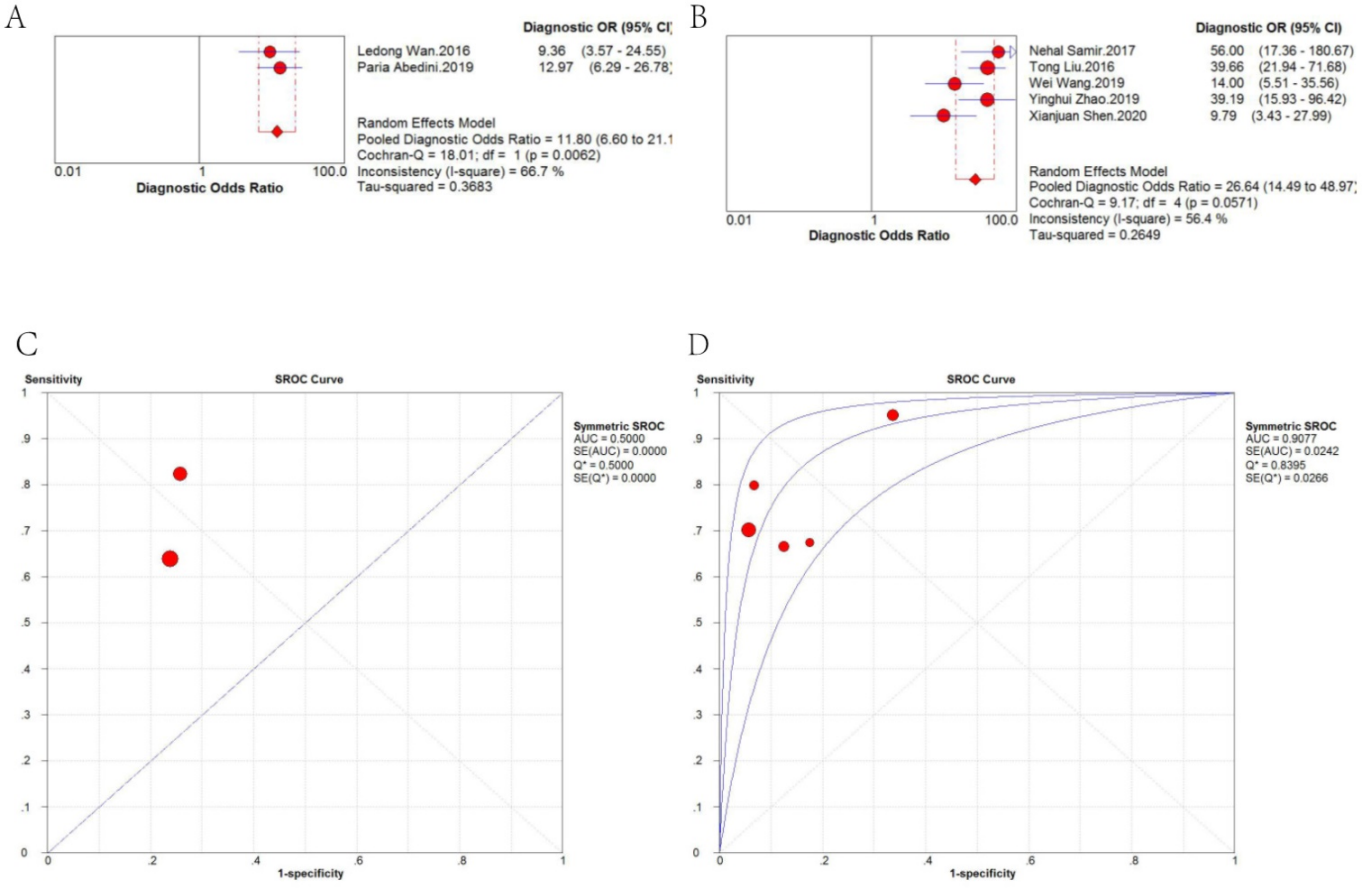
DOR and sROC of (A) plasma-based and (B) serum-based detection of lncRNAs for the diagnosis of CRC.

Therefore, we further analyzed the effect of sample type on diagnosis (*Figure 5*). A random-effects model was applied because there was significant heterogeneity (*I*^2^ = 63.6% and *I*^2^ = 80.6%, respectively). The stratified analysis showed that the performance of blood-based detection was significantly superior to that of tissue-based detection (AUC: 0.8820 *vs.* 0.8203; pooled DOR: 18.57 *vs.* 10.47). Blood-based tests were then divided into two categories, plasma-based and serum-based lncRNA testing. The results in *Figure 6* revealed that the AUC of serum-based detection was 0.9077, pooled DOR: 26.64, and plasma-based detection was 0.5000, pooled DOR: 11.80.

### Publication bias

**Figure 7:**
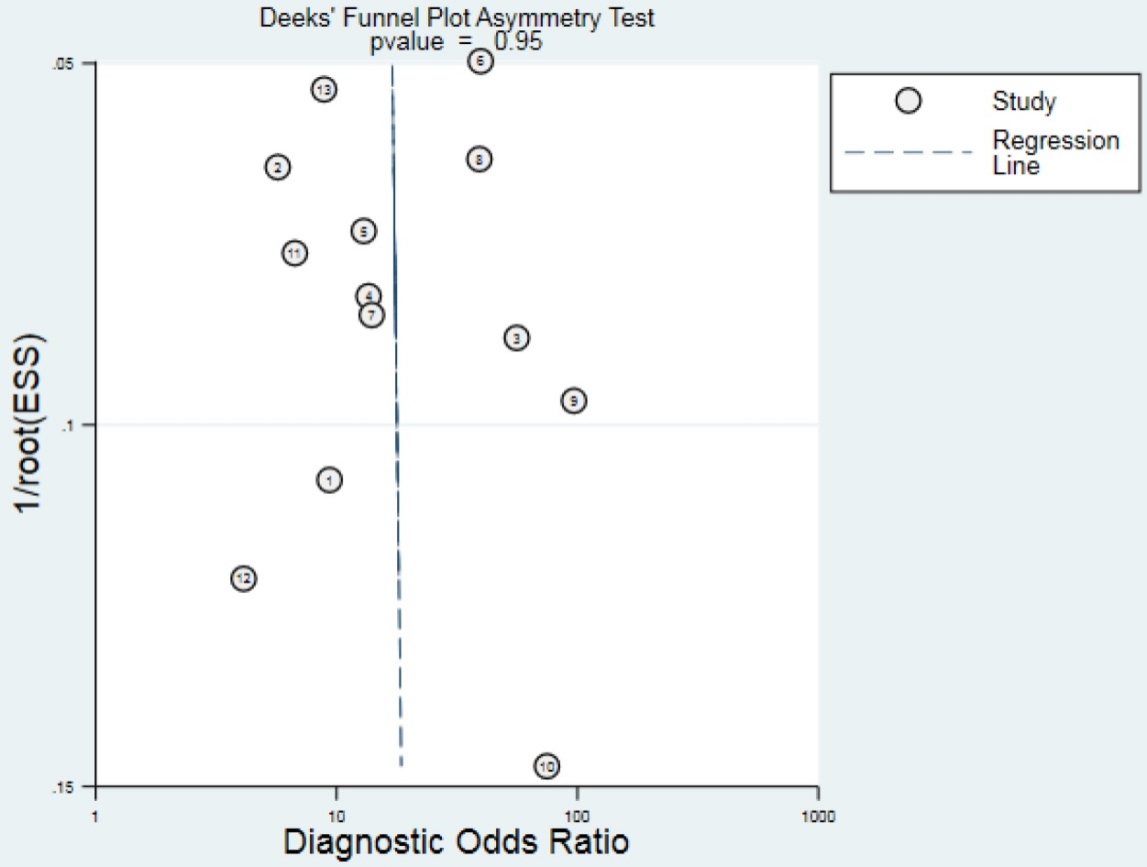
Funnel plot for the assessment of potential publication bias of the diagnostic studies.

Deeks' funnel plot asymmetry test was performed to check publication bias in this meta-analysis. The result presented in *Fig. 7* showed no evidence of publication bias (*P* = 0.95) existed for diagnostic analyses.

## Discussion

Colorectal cancer is a major cause of cancer mortality worldwide. Early diagnosis greatly increases the chances of successful treatment and improves the cancer outcomes, potentially reducing mortality from cancer 30. LncRNAs are characterized as a group of endogenous RNAs that have no protein-encoding function 31, 32. Recent studies have suggested that the expression of lncRNAs in CRC might be involved in cancer development, invasion, metastasis, prognosis 33-36. Statistically significant differences in the expression of lncRNAs between precancerous lesions and early-stage cancer have been found in previous studies 37-39. However, there is no relevant systematic review and meta-analyses focused on the expression of lncRNAs in CRC diagnosis. In this meta-analysis, we systematically analyzed the relationship between the expression of lncRNAs and the early diagnosis of CRC.

The included studies were mainly published between 2016 to 2020 and deemed to be of high quality according to the QUADAS-2. The results of our meta-analysis showed that the expression of LncRNAs was significantly associated with tumor size, differentiation, TNM stage, metastasis, levels of CA19-9 and CEA. It indicates that the dysregulated expression of lncRNAs is implicated in the progression of CRC and might act as potential biomarkers for the early diagnosis of CRC.

The ROC curve is a comprehensive index, which reflects the sensitivity and specificity of continuous variables. The pooled AUC of lncRNAs indicated that 86.29% of randomly chosen CRC patients had higher or lower levels of lncRNAs than normal controls. The pooled DOR is also an important indicator that facilitates formal meta-analysis of studies on diagnostic test performance. In the present study, a pooled DOR of 14.20 (higher than 1.0) was obtained, suggesting that detection of lncRNA is a powerful predictive biomarker for CRC diagnosis.

Fifteen types of lncRNAs with a different expression status in CRC were included in this review. And the meta-regression test further showed that different kind of samples for lncRNA detection was probably the source of heterogeneity (*P* = 0.0339, RDOR = 1.69, 95%CI: 1.05 - 2.72). Therefore we conducted subgroup analyses to compare the difference. Stratified analyses based on sample type showed that blood-based lncRNA analysis was of higher diagnostic efficacy than tissue-based analysis (AUC: 0.8820 *vs.* 0.8203; pooled DOR: 18.57 *vs.* 10.47). Moreover, serum-based lncRNA testing achieved higher diagnostic efficacy than plasma-based analysis (AUC: 0.9077 *vs.* 0.5000; pooled DOR: 26.64 *vs.*11.80), suggesting that serum-based detection is more recommended.

The Deeks' funnel plot asymmetry test didn't reveal obvious publication bias for the diagnostic meta-analyses, suggesting that results from pooled data analysis were reliable. When taking into consideration the stable structures and easy detection, lncRNAs have the potentials to serve as novel, easily attainable biomarkers for the diagnosis of CRC.

Several limitations should be acknowledged in this systematic review and meta-analysis. Firstly, only 15 types of lncRNAs were included and they were analyzed based on different quality control standards, which might have generated potential heterogeneity. Secondly, the small sample size in sub-group analyses led to low statistical power and undermined the strength of the evidence of this systematic review. Thirdly, the majority of patients included in our study were Asians, potential ethnic-related differences in the expression of lncRNAs might restrict the applicability of our findings to other races. Finally, the clinical value of lncRNAs in CRC patients might have been exaggerated because the studies with positive results were more likely to be published than those with negative results.

## Conclusion

In summary, this is the first meta-analysis to evaluate the clinical value of lncRNAs in the diagnosis of colorectal cancer. The results of our meta-analysis reveal that lncRNAs are promising diagnostic biomarkers in patients with CRC. Blood-based lncRNA analysis is of higher diagnostic accuracy than tissue-based testing. Moreover, serum-based lncRNA testing shows higher diagnostic efficacy than plasma-based analysis. However, considering the mentioned limitations above, a larger number of clinical trials are still needed to further verify the findings and confirm the clinical application of lncRNAs in the diagnosis of CRC. Further prospective studies on the role of each specific lncRNA in the early diagnosis of CRC are also warranted in the future.

## Figures and Tables

**Table 1:** Main characteristics of the meta-analysis for diagnostic performance and clinicopathologic association of lncRNAs in CRC patients

First author	Publication Year	LncRNA	Expression	Patient number	Control number	Sample size	Sample type	Detection method	Cut-Off Value	AUC	95%CI	SEN%	SPE%
Meng Xu	2020	HANR	up	165	165	330	tissue	qRT-PCR	meidan	0.82	0.775-0.865	60.0	82.0
Xianjuan Shen	2020	DANCR	up	40	40	80	serum	qRT-PCR	1.994	0.747	0.638-0.857	67.5	82.5
Baolian Song	2019	lnc RNA-1	up	77	30	107	tissue	qRT-PCR	NA	0.788	NA	83.7	64.2
Paria Abedini	2019	lnc-ATB	up	74	74	148	plasma	qRT‐PCR	2.5000	0.780	0.811-0.940	82.0	75.0
Peng Jiang	2019	NKILA	down	70	140	210	tissues	qRT-PCR	4.5000	0.839	NA	82.9	72.9
Wei Wang	2019	MEG3	down	126	48	174	serum	qRT-PCR	NA	0.798	0.730-0.866	66.7	87.5
Yinghui Zhao	2019	LINC02418	up	125	125	250	serum	qRT-PCR	2.9590	0.898	0.864-0.935	95.2	66.4
Q.-G. LI	2019	lnc-DILC	down	174	174	348	tissue	RT-qPCR	median	0.826	NA	78.0	71.0
Yueqiong Lao	2018	ENST00000455974	up	45	66	111	tissues	qRT-PCR	0.0005	0.899	0.821-0.977	95.6	81.2
Jiangtao Yu	2018	SLCO4A1-AS1	up	50	15	65	tissue	qRT-PCR	median	0.924	0.852-0.996	92.2	87.0
Yeshuo Ma	2018	RP1-85F18.6	up	34	34	68	tissue	RT-qPCR	median	0.651	0.516‐0.785	55.9	76.5
Nehal Samir	2017	LncRNA-RP11-909B2	down	70	60	130	serum	qRT-PCR	0,1800	0.867	0.807-0.925	80.0	93.3
Ledong Wan	2016	HOTAIRM1	down	150	101	251	plasma	qRT-PCR	0.0030	0.780	0.708-0.841	64.0	76.5
Tong Liu	2016	CRNDE-h	up	148	320	468	serum	RT-qPCR	0.0200	0.892	0.860-0.918	70.3	94.4
Debing Shi	2014	RP11-462C24.1	down	86	86	172	tissue	qRT-PCR	mean	0.778	NA	69.4	73.9

LncRNA: long non-coding RNA; AUC: area under the curve; SEN: sensitivity; SPE: specificity; NA: not available.

**Table 2:** Associations between lncRNAs and clinicopathological parameters in CRC patients

Clinicopathological parameters	Combined* P* value	X^2^ value	Enrolled studies
Age	0.55071502	26.406427	14
Gender	0.25576268	30.290211	13
Tumor location	0.75194819	11.883670	8
Tumor size^*^	0.00000019	73.442684	11
differentiation	0.03842262	27.284946	8
Lymphatic metastasis^*^	0.00000114	88.481443	11
Distal metastasis	0.02710721	23.073642	6
TNM stage^*^	0.00000002	83.733916	12
Depth of invasion	0.01862524	18.368803	4
CEA level^*^	0.00073771	26.892954	4
CA19-9 level	0.01445876	19.075940	4

*P < 0.01.

**Table 3:** Analysis of Diagnostic Threshold in weighted regression (inverse variance)

Variance	Coefficient	Standard Error	T	*P* value
a	2.693	0.237	11.383	0.0000
b	0.178	0.204	0.871	0.3995

Spearman correlation coefficient: 0.314, *p*-value = 0.254; Tau-squared estimate = 0.6115 (convergence is achieved after 6 iterations); Restricted maximum likelihood estimation (REML); No. studies = 15.

**Table 4:** Meta-regression (inverse variance weights)

Variance	Coefficient	Standard Error	*P* value	RDOC	[95% CI]
Cte.	2.823	1.2690	0.0503	----	----
S	0.228	0.1873	0.2517	----	----
Size	-0.394	0.6150	0.5366	0.67	(0.17 - 2.66)
Sample	0.482	0.2326	0.0651	1.62	(0.96 - 2.72)
Expression	-0.393	0.4413	0.3951	0.67	(0.25 - 1.80)

Tau-squared estimate = 0.3808 (convergence is achieved after 11 iterations); Restricted maximum likelihood estimation (REML); No. studies = 15.

**Table 5:** Meta-regression (remove size)

Variance	Coefficient	Standard Error	*P* value	RDOC	[95% CI]
Cte.	2.481	0.9584	0.0293	----	----
S	0.198	0.2100	0.3694	----	----
Sample	0.501	0.2261	0.0488	1.65	(1.00 - 2.71)
Expression	-0.279	0.3937	0.4937	0.76	(0.32 - 1.80)

Tau-squared estimate = 0.3560 (convergence is achieved after 10 iterations); Restricted maximum likelihood estimation (REML); No. studies = 15.

**Table 6:** Meta-regression (remove expression)

Variance	Coefficient	Standard Error	*P* value	RDOC	[95% CI]
Cte.	1.729	0.4298	0.0017	----	----
S	0.268	0.1721	0.1453	----	----
Sample	0.524	0.2187	0.0339*	1.69	(1.05 - 2.72)

Tau-squared estimate = 0.3297 (convergence is achieved after 9 iterations); Restricted maximum likelihood estimation (REML); No. studies = 15.
